# Number of prior live births is associated with higher arterial stiffness but not its change in older women: the atherosclerosis risk in communities study

**DOI:** 10.3389/fcvm.2023.1172828

**Published:** 2023-05-23

**Authors:** Alison N. Bonner, Shantal Jayawickreme, Angela M. Malek, Catherine J. Vladutiu, Clare Oliver-Williams, Yamnia I. Cortés, Hirofumi Tanaka, Michelle L. Meyer

**Affiliations:** ^1^ Medical Doctorate Program, University of North Carolina School of Medicine, Chapel Hill, NC, United States; ^2^ Department of Public Health Sciences, Medical University of South Carolina, Charleston, SC, United States; ^3^ Department of Obstetrics & Gynecology, University of North Carolina at Chapel Hill, Chapel Hill, NC, United States; ^4^ Department of Health Sciences, University of Leicester, Leicester, United Kingdom; ^5^ Central Bedfordshire Council, Chicksands, Bedfordshire, United Kingdom; ^6^ School of Nursing, University of North Carolina at Chapel Hill, Chapel Hill, NC, United States; ^7^ Department of Kinesiology and Health Education, University of Texas at Austin, Austin, TX, United States; ^8^ Department of Emergency Medicine, University of North Carolina at Chapel Hill, Chapel Hill, NC, United States

**Keywords:** arterial stiffness (AS), parity, womens health, ARIC atherosclerosis risk in communities study, longitudinal

## Abstract

**Introduction:**

Although studies have demonstrated a J-shaped association between parity and cardiovascular disease (CVD), the association with arterial stiffness is not fully understood.

**Methods:**

We examined the association between parity and carotid-femoral pulse wave velocity (cfPWV), a measure of central arterial stiffness. We conducted a longitudinal analysis of 1220 women (mean age 73.7 years) who attended the Atherosclerosis Risk in Communities Study visit 5 (2011-2013). At visit 2 (1990-1992), women self-reported parity (number of prior live births), which we categorized as: 0 (never pregnant or pregnant with no live births); 1-2 (referent); 3-4; and 5+ live births. Technicians measured cfPWV at visit 5 (2011-2013) and visit 6 or 7 (2016-2019). Multivariable linear regression modeled the associations of parity with visit 5 cfPWV and cfPWV change between visit 5 and 6/7 adjusted for demographics and potential confounding factors.

**Results:**

Participants reported 0 (7.7%), 1-2 (38.7%), 3-4 (40.0%), or 5+ (13.6%) prior live births. In adjusted analyses, women with 5+ live births had a higher visit 5 cfPWV (*β*=50.6 cm/s, 95% confidence interval: 3.6, 97.7 cm/s) than those with 1-2 live births. No statistically significant associations were observed for other parity groups with visit 5 cfPWV or with cfPWV change.

**Discussion:**

In later life, women with 5+ live births had higher arterial stiffness than those with 1-2 live births, but cfPWV change did not differ by parity, suggesting women with 5+ live births should be targeted for early primary prevention of CVD given their higher arterial stiffness at later-life.

## Introduction

Cardiovascular disease (CVD) is the leading cause of death for women in the United States (U.S.), resulting in one third of all female deaths—approximately 400,000 deaths per year (1). While more than 26 million adults in the U.S. have diagnosed CVD (2), the level of undiagnosed CVD is significant, with 20% of myocardial infarctions going undetected at the time of occurrence (3). Notably, women are less likely than men to receive preventive guidance and treatments, such as statins, aspirin, and therapeutic lifestyle changes, which may lead to increased prevalence of subclinical CVD in women (1). In addition to traditional risk factors, certain CVD risk factors are unique to women, including pregnancy-related factors (4–8). Studies have shown that pregnancy-related factors, such as parity (defined as the number of prior live births) (6, 9), are associated with later-life CVD, as measured by the incidence of CVD and CVD events. Previous analyses have observed a J-shaped association between parity and incident CVD, with the lowest CVD risk among women who had 2 live births (6, 10). A recent analysis in the Atherosclerosis Risk in Communities (ARIC) Study, a population-based cohort examining the etiology of atherosclerosis, found that women with 5+ live births had a greater risk of CVD over a 30-year follow-up even after adjusting for socioeconomic factors and pregnancy complications (10). Since 2011, the American Heart Association's CVD prevention guidelines include an assessment of obstetric history when evaluating a woman's cardiovascular risk, emphasizing the role of pregnancy and pregnancy-related events in morbidity and mortality from CVD, and highlighting the importance of targeting younger women for primary prevention (5).

While associations between parity and later-life CVD events have been well established, data linking parity to subclinical vascular disease are limited. Arterial stiffness, a measure of structural and functional changes in the arterial walls can be used to assess preclinical stages of vascular disease and is a strong predictor of CVD events and mortality (11). Arterial stiffness is most commonly expressed by pulse wave velocity (PWV), which measures the transit time of the forward pulse wave between two arterial sites, such as carotid-femoral PWV (cfPWV) and brachial-ankle PWV (baPWV). Given its association with increased CVD risk, arterial stiffness is an active area of research for CVD risk stratification (6). Yet, the association between parity and arterial stiffness is not fully understood. Only one study to date has examined the relationship between parity and PWV, in which it was shown that higher parity was associated with higher baPWV, but not after adjusting for potential confounding factors (12). Thus, further studies are needed to characterize the association between parity and arterial stiffness. Understanding the mechanism by which childbearing increases CVD could help to direct preventive efforts in women earlier in life based on their obstetric history, with the goal of reducing CVD incidence in women.

Therefore, we aimed to evaluate the association between parity and central arterial stiffness in a population of older-aged women from diverse backgrounds across the U.S. We hypothesized that women who were never pregnant or pregnant with no live births and women with 5+ live births would have higher cfPWV and greater cfPWV change over time compared to women with 1–2 live births.

## Materials and methods

### Study population

The ARIC study is a population-based, longitudinal study of 15,792 participants aged 45–64 years at the time of their enrollment in 1987–1989 from the following four U.S. communities: Forsyth County, North Carolina; Jackson, Mississippi; Minneapolis, Minnesota; and Washington County, Maryland. Details of the baseline visit have been previously described (13). This analysis includes 2,686 participants with cfPWV measured at visit 5 (2011–2013) and either visit 6 (2016–2017) or visit 7 (2018–2019). Due to PWV measurement quality concerns, we excluded participants with body mass index (BMI) >40 kg/m^2^ at either visit (*n* = 65) or missing BMI (*n* = 1), major arrhythmias (Minnesota code 8-1-3, 8-3-1, and 8-3-2; *n* = 47), Minnesota code 8-1-2 with low quality PWV waveforms (*n* = 12), self-reported aortic revascularization (*n* = 20), echocardiographic evidence of aortic stenosis or moderate or greater aortic regurgitation (*n* = 15), and missing covariates of interest (*n* = 11) (14). Due to small sample sizes, the following participants were excluded from all analyses: persons who self-identified as Asian or American Indian from any site (*n* = 9), African American from the Minnesota and Maryland sites (*n* = 8), and African American from the North Carolina site (*n* = 11). For this analysis, we further excluded men (*n* = 1,091), participants missing cfPWV values at both visit 5 and visit 6 or 7 (*n* = 165), and those missing parity information (*n* = 11). The final analytic sample included 1,220 women after exclusions. Participants provided written informed consent and ARIC was approved by the Institutional Review Boards at all field centers and other study agencies.

### Measures

Before the study visit, participants were asked to not consume food or drinks, and to refrain from tobacco use and vigorous physical activity after midnight prior to the clinic visit or for 8 h prior to the visit. At visit 1, participants completed interviewer-administered questionnaires to assess demographic information, including age, race, and education level. Race and study center were defined as a combined variable for the analysis. At visit 5, participants completed another questionnaire to determine cigarette smoking status (current, former, never). Anthropometric and cardiometabolic measures, including BMI, waist circumference, blood pressure, mean arterial pressure, and heart rate were measured prior to PWV measurements. Blood samples were obtained following a standardized venipuncture protocol and shipped to ARIC central laboratories where assays for total cholesterol, high-density lipoprotein (HDL) cholesterol, triglycerides, and fasting glucose concentrations were performed. Low-density lipoprotein (LDL) cholesterol was calculated using the Friedewald equation (15). Glycated hemoglobin (HbA1c) was measured in EDTA whole blood on the Tosoh high-performance liquid chromatography Glycohemoglobin Analyzer (Tosoh Medics, Inc., San Francisco, CA) calibrated with standard values derived by the National Glycohemoglobin Standardization Program.

Body weight, measured to the nearest 0.1 kilogram, and height recorded to the nearest centimeter, were used to calculate BMI (weight/height^2^). Three seated blood pressure measurements were obtained after a five-minute rest using an oscillometric automated sphygmomanometer (Omron HEM-907 XL, Omron Co. Ltd., Kyoto, Japan) and the average of the last two measurements was used. Hypertension was defined as systolic blood pressure (SBP) ≥ 140 mmHg, diastolic blood pressure (DBP) ≥ 90 mmHg (16), or anti-hypertensive medication use. Diabetes mellitus was defined as fasting glucose ≥126 mg/dl, non-fasting glucose ≥200 mg/dl, anti-diabetic medication use, or self-reported physician diagnosis of diabetes mellitus.

### Reproductive factors

At the baseline ARIC study visit, study staff administered a questionnaire asking women to report the total number of prior live births (parity). At visit 3, women were asked about age at menarche and whether they had ever been pregnant (gravidity). Using these data, we categorized women into four groups: 0 live births (never pregnant or pregnant with no live births), 1–2 live births, 3–4 live births, and 5+ live births.

### Pulse wave velocity

At visits 5, 6, and 7, technicians measured cfPWV using the same standardized protocol. Details of the PWV methodology for the ARIC study have been reported (14). Briefly, technicians measured PWV using the automated waveform analyzer VP-1000 Plus (Omron Co., Ltd., Kyoto, Japan) (17) after participants were supine for 5–10 min. Carotid and femoral arterial pressure waveforms were acquired by applanation tonometry sensors on the left common carotid artery and left common femoral artery. PWV was calculated as distance divided by transit time. Distance for cfPWV was measured with a segmometer (Rosscraft, Surray, Canada) and calculated as the carotid to femoral distance minus the distance between the suprasternal notch to carotid. Technicians obtained at least two measurements and results were averaged (18). Repeat visits were conducted for a subset of participants at each field center approximately 4–8 weeks after visit 5 only (*n* = 79; mean age 75.7 years; 46 females). At visit 5, the intra-class correlation coefficient and 95% confidence interval (95% CI) for single cfPWV measurements were 0.70 (0.59, 0.81).

### Missing data

Participants missing information on PWV or any covariates were excluded from analysis.

### Statistical analysis

Author MLM had full access to all the data in the study and takes responsibility for its integrity and the data analysis. Participant characteristics were compared by parity categories (0 live births, 1–2 live births, 3–4 live births, or 5+ live births). Categorical variables were presented as number and percentage, while continuous variables were presented as mean and standard deviation.

### Continuous outcomes

We winsorized cfPWV values that were beyond the 1% and 99% of the distribution for cfPWV at visit 5 (*n* = 23) and visit 6 or 7 (*n* = 21). Change in cfPWV was calculated as cfPWV at visit 6 or 7 minus cfPWV at visit 5, and these values were also winsorized (*n* = 20). We used analysis of covariance (ANCOVA) to estimate the adjusted means of cfPWV in each group. Multivariable linear regression modeled the associations of parity with cfPWV at visit 5 and change in cfPWV from visit 5 to 6/7. For the regression analyses, we adjusted for variables that were associated with both change in cfPWV and parity and variables known in the literature to be associated with both cfPWV and parity. Covariates included age, race-center (race by study center), education, and cardiometabolic factors measured at visit 5, including BMI, heart rate, mean arterial pressure (MAP), hypertension medication use, and diabetes mellitus. For cfPWV change, we additionally adjusted for years between visit 5 (2011–2013) and visit 6 (2016–2017) or visit 7 (2018–2019), with a mean time of 5.7 years. For the regression analyses, we estimated the association between parity and cfPWV and change in cfPWV in 3 models: model 1 adjusted for years between visit 5 and 6 or 7, model 2 additionally adjusted for age, race-center, and education, and model 3 additionally adjusted for BMI, heart rate, MAP, hypertension medication use, and diabetes mellitus.

### Categorical outcomes

Multivariable logistic regression was also used to evaluate associations of parity with higher cfPWV at visit 5 and elevated cfPWV change, both defined as >75th percentile of the distribution. Covariates and adjusted models were the same as those used in the regression analyses of continuous outcomes. *P*-values were two-sided with statistical significance of *P* < 0.05 (SAS, version 9.2, SAS Institute, Inc., Cary, NC).

## Results

### Participant characteristics

Women were on average 73.7 years of age at visit 5, and 22% identified as African American. Participants reported having 0 live births (7.7%), 1–2 (38.7%), 3–4 (40.0%), or 5+ (13.6%) live births (Table 1). On average, women with 5+ live births were more likely than women in the other groups to be African American, have less than a high school education, a higher BMI, and a higher prevalence of diabetes and hypertension. They were also more likely to be younger at the birth of their first child.

**Table 1:** Participant characteristics by parity categories at ARIC visit 5 (*n* = 1,220).

	Number of prior live births				
Variable	Overall	0^†^	1–2	3–4	5+
*N* (%)	1,220 (100%)	94 (7.7%)	472 (38.7%)	488 (40.0%)	166 (13.6%)
Age*, years (SD)	73.7 (4.3)	73.6 (4.2)	73.1 (4.1)	73.6 (4.1)	75.6 (4.6)
Race and center, *n* (%)					
African American, Jackson, MS	267 (21.9%)	13 (13.8%)	96 (20.3%)	90 (18.4%)	68 (41.0%)
White, Forsyth County, NC	203 (16.6%)	16 (17%)	106 (22.5%)	69 (14.1%)	12 (7.2%)
White, Minneapolis, MN	409 (33.5%)	29 (30.9%)	141 (29.9%)	193 (39.5%)	46 (27.7%)
White, Washington County, MD	341 (28.0%)	36 (38.3%)	129 (27.3%)	136 (27.9%)	40 (24.1%)
Education, *n* (%)					
<High School	128 (10.5%)	7 (7.4%)	35 (7.4%)	35 (7.2%)	51 (30.7%)
High School	445 (36.5%)	35 (37.2%)	162 (34.3%)	186 (38.1%)	62 (37.3%)
>High School	647 (53.0%)	52 (55.3%)	275 (58.3%)	267 (54.7%)	53 (31.9%)
Body mass index (BMI), kg/m^2^ (SD)	27.6 (4.5)	26.5 (4.7)	27.4 (4.5)	27.6 (4.3)	28.6 (4.5)
Systolic blood pressure, mmHg (SD)	129.6 (16.7)	129.7 (16.6)	129.2 (16.8)	129.4 (16.3)	131.3 (17.8)
Diastolic blood pressure, mmHg (SD)	66.7 (10.1)	66.8 (10.5)	66.8 (10.0)	66.6 (9.9)	66.3 (10.6)
Mean arterial pressure, mmHg (SD)	87.6 (11.0)	87.7 (11.2)	87.6 (10.9)	87.6 (10.7)	88.0 (11.8)
Heart rate, beats per minute (SD)	62.0 (9.1)	61.0 (7.8)	61.8 (8.8)	62.4 (9.4)	61.9 (9.7)
Blood pressure lowering medication, *n* (%)	823 (67.5%)	57 (60.6%)	306 (64.8%)	332 (68.0%)	128 (77.1%)
Smoking status, *n* (%)					
Current	58 (4.8%)	3 (3.2%)	23 (4.9%)	26 (5.4%)	6 (3.7%)
Former	501 (41.4%)	46 (49.5%)	196 (41.7%)	199 (41.2%)	60 (36.6%)
Never	604 (49.9%)	41 (44.1%)	235 (50.0%)	240 (49.7%)	88 (53.7%)
Unknown smoking history	47 (3.9%)	3 (3.2%)	16 (3.4%)	18 (3.7%)	10 (6.1%)
Type 2 diabetes mellitus, *n* (%)	269 (22.0%)	13 (13.8%)	100 (21.2%)	104 (21.3%)	52 (31.3%)
Reproductive years, years (SD)	33.3 (6.7)	32.8 (7.7)	33.1 (6.8)	33.8 (6.4)	32.8 (6.7)
Age when first child born, years (SD)	22.5 (4.0)	–	23.9 (4.3)	22.1 (3.2)	19.9 (3.0)
Carotid-femoral pulse wave velocity (cfPWV) cm/s (SD), visit 5	1,106.4 (280.8)	1,094.0 (259.7)	1,077.8 (261.5)	1,103.3 (269.5)	1,204.1 (350.7)
Carotid-femoral pulse wave velocity (cfPWV) cm/s (SD), visit 6/7	1,251.8 (337.2)	1,262.6 (394.7)	1,228.0 (313.5)	1,238.3 (327.3)	1,353.4 (378.1)

SD, Standard Deviation; MD, Maryland; MN, Minnesota; MS, Mississippi; NC, North Carolina.

* Age range: 67–88 years.

^†^ Among the never pregnant or no live births group, 10 (10.6%) women were pregnant but had no live births.

### Continuous outcomes

**Figure 1:**
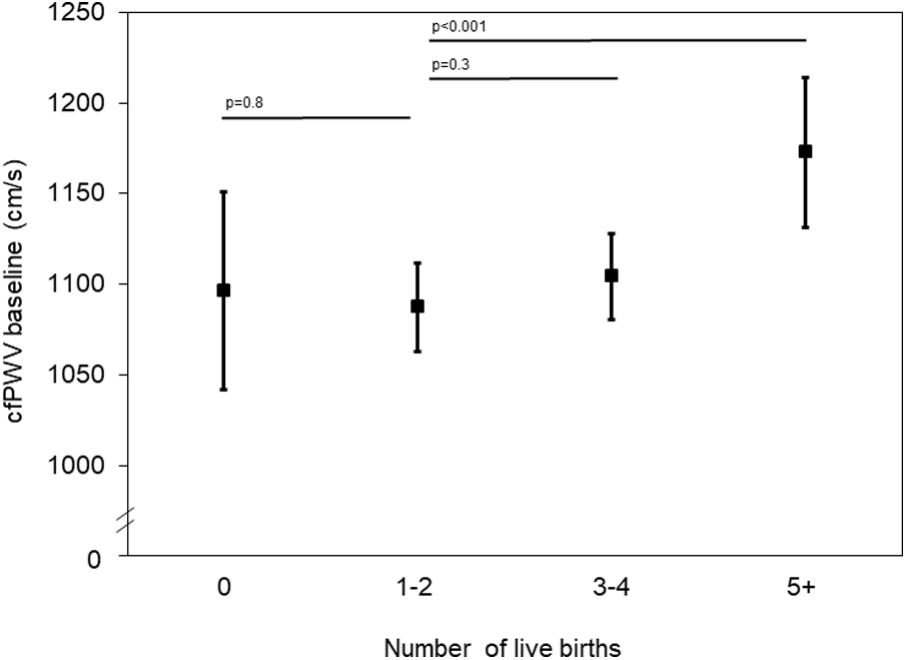
Carotid-femoral pulse wave velocity (cfPWV) measurements at visit 5 by parity group, adjusted for age.

The age-adjusted mean of cfPWV at visit 5 compared by parity groups demonstrates a J-shaped curve with the lowest measurements in women with 1–2 live births (Figure 1), although the 95% CIs overlapped. Women with 5+ live births had higher cfPWV measurements when compared with women with 1–2 live births in the unadjusted model as well as the fully adjusted model (Model 3: *β *= 50.6, 95% CI: 3.6, 97.7) (Table 2). No other parity groups in any of the models demonstrated a statistically significant difference in cfPWV when compared to women with 1–2 live births.

**Table 2:** Association of parity and carotid-femoral pulse wave velocity (cfPWV) at ARIC visit 5 (*n* = 1,220).

		0 live births		1–2 live births	3–4 live births		5+ live births	
		*β*	(95% CI)	REFERENT	*β*	(95% CI)	*β*	(95% CI)
cfPWV at visit 5 (cm/s)	Model 1^†^	16.2	(−45.4, 77.9)		25.5	(−9.7, 60.8)	126.3	(77.1, 175.6)*
	Model 2^‡^	14.1	(−45.1, 73.3)		18.1	(−16.0, 52.1)	46.0	(−4.3, 96.2)
	Model 3^§^	16.9	(−38.6, 72.3)		14.3	(−17.5, 46.2)	50.6	(3.6, 97.7)*
Elevated cfPWV at visit 5 (>75th percentile)^‖^		OR	(95% CI)		OR	(95% CI)	OR	(95% CI)
	Model 1^†^	1.56	(0.96, 2.52)		0.98	(0.73, 1.32)	1.09	(0.73, 1.64)
	Model 2^‡^	1.59	(0.97, 2.70)		1.07	(0.79, 1.45)	1.07	(0.70, 1.64)
	Model 3^§^	1.64	(1.00, 2.70)		1.07	(0.79, 1.45)	1.04	(0.67, 1.60)

*β*, unstandardized coefficient in original units; CI, confidence interval; OR, odds ratio.

* Statistically significant by 95% CI.

^†^ Model 1: unadjusted.

^‡^ Model 2: age, race-center, and education.

^§^ Model 3: Model 2 + visit 5 body mass index, heart rate, mean arterial pressure, hypertension medication use, and diabetes mellitus.

^‖^ >75th percentile ≥1,252.0 cm/s, *n* = 304, range: 1,253.5–2,124.0 cm/s.

≤75th percentile: *n* = 916, range: 529.0–1,252.0 cm/s.

**Figure 2:**
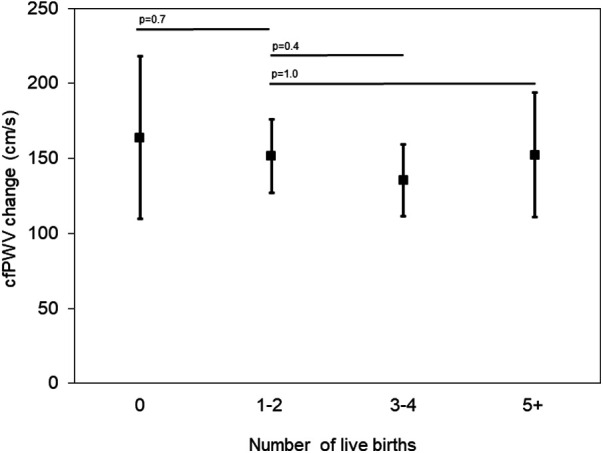
Carotid-femoral pulse wave velocity (cfPWV) change from visit 5 to visit 6/7 by parity group, adjusted for age and years between visit 5 and visits 6 or 7.

Compared to the referent group of women with 1–2 live births, the adjusted mean cfPWV change from visit 5 to 6/7 was lowest in women with 3–4 live births and higher in women with 0 live births and in women with 5+ live births, but these differences were not statistically significant as indicated by the 95% CI (means adjusted for age and years between visit 5 and 6 or 7; Figure 2). The average cfPWV change ranged from 135.5 cm/s in women with 3–4 live births to 163.9 cm/s in women with 0 live births. In multivariable regression analyses, cfPWV change from visit 5 to visit 6/7 (average follow-up time 5.7 years) compared to women with 1–2 live births was not significantly different in any of the other parity groups (Table 3).

**Table 3:** Association of parity and carotid-femoral pulse wave velocity (cfPWV) change from ARIC visit 5 to visit 6/7 (*n* = 1,220).

		0 live births		1–2 live births	3–4 live births		5+ live births	
		*β*	(95% CI)	REFERENT	*β*	(95% CI)	*β*	(95% CI)
cfPWV change (cm/s)	Model 1*	12.9	(−46.5, 72.4)		−15.3	(−49.3, 18.7)	3.5	(−44.0, 51.0)
	Model 2^†^	10.4	(−48.2, 69.0)		−5.2	(−38.9, 28.6)	5.6	(−44.2, 55.4)
	Model 3^‡^	14.7	(−43.9, 73.2)		−1.9	(−35.5, 31.7)	5.9	(−43.8, 55.6)
Elevated cfPWV change (>75th percentile)^§^		OR	(95% CI)		OR	(95% CI)	OR	(95% CI)
	Model 1*	1.55	(0.96, 2.51)		0.97	(0.72, 1.31)	1.09	(0.72, 1.63)
	Model 2^†^	1.58	(0.97, 2.58)		1.05	(0.77, 1.43)	0.99	(0.64, 1.54)
	Model 3^‡^	1.64	(1.00, 2.68)		1.08	(0.79, 1.46)	1.00	(0.64, 1.56)

*β*, unstandardized coefficient in original units; CI, confidence interval; OR, odds ratio.

* Model 1 adjusted for years between visit 5 and visit 6/7.

^†^ Model 2 adjusted for Model 1 + age, race-center, and education.

^‡^ Model 3 adjusted for Model 2 + visit 5 body mass index, heart rate, mean arterial pressure, hypertension medication use, and diabetes mellitus.

^§^ >75th percentile: >292.0 cm/s, *n* = 303, range: 293.5 to 961.5 cm/s.

≤75th percentile: *n* = 917, range −680.0 to 292.0 cm/s.

### Categorical outcomes

Similar to the analysis of cfPWV as a continuous measurement, we did not observe a significant association between parity and higher categorical cfPWV (>75th percentile). However, those with 0 live births had higher odds of elevated cfPWV [odds ratio (OR) = 1.64, 95% CI: 1.00, 2.70], but this association was not statistically significant. Analysis of categorical change in cfPWV was also consistent with the continuous results, showing women with 0 live births were more likely to have elevated progression of cfPWV (>75th percentile) after adjusting for years between visit 5 and 6 or 7, age, race-center, education, heart rate, MAP, BMI, hypertension medication use, and diabetes mellitus (OR = 1.64, 95% CI: 1.00, 2.68), although this association was not statistically significant.

## Discussion

Using data from the ARIC study, which includes a population of older-aged women with diverse backgrounds from four communities in the U.S., we found that women with 5+ live births had higher measures of cfPWV at ARIC visit 5, with average cfPWV measures 50.6 cm/s higher than the referent group of women with 1–2 live births. The average cfPWV change ranged from 135.5 cm/s in women with 3–4 live births to 163.9 cm/s in women with no live births, though the difference between parity groups was not statistically significant.

Currently, only one other study has specifically examined the relationship between parity and PWV. Using data from the Study of Women's Health Across the Nation (SWAN), this prior study also found that higher parity (3+ live births) was associated with higher baPWV, though the association was not significant after adjusting for potential confounding factors, including demographic and cardiometabolic factors similar to those used in our study (12). However, baPWV measures both central and peripheral arterial stiffness, whereas our study uses cfPWV, a specific measure of central arterial stiffness (19). Additionally, the current study focused on older participants (73.7 years vs. 60.2 years), and has a larger sample size (1,220 vs. 964) than SWAN, which provided enough data to assess parity groups of 3–4 and 5+ live births (12).

Cross-sectional studies have shown that every 1 m/s elevation in cfPWV is associated with a 14% increased risk of a cardiovascular event (includes both cardiovascular deaths and nonfatal events) and a 15% increased risk of mortality by both cardiac and non-cardiac causes (11). Thus the increased cfPWV observed in the 5+ live births group in our study is consistent with previous studies showing an increased CVD risk in women with higher parity, including the Framingham Heart Study (6+ live births), the National Health and Nutrition Examination Survey I, Epidemiologic Follow-up Study (6+ live births), and the SWAN study (3+ live births) (6, 9, 10, 12, 20). Compared to the referent group, visit 5 cfPWV was statistically different only among women with 5+ live births. However; cfPWV was a slightly higher among women with no live births compared to the referent group, consistent with the association between parity and CVD demonstrated in prior studies, though there was a wide confidence interval in the current study (6, 9, 10).

Higher parity is also associated with a number of cardiometabolic factors that could cause increased arterial stiffness later in life, including increased body weight (21), increased waist circumference (21), elevated blood pressure (22), and insulin resistance (22). These associations are consistent with those observed in the ARIC study cohort, where we found women with 5+ live births had higher BMI, waist circumference, and prevalence of hypertension and diabetes mellitus, than women in the other parity groups. Thus, we included these factors as potential confounders in the analysis. Social factors, including race, socioeconomic status, and other unmeasured factors, are associated with differences in CVD risk and could also affect our results (23, 24). We adjusted for these social factors in the regression analyses (using education level as a representation of socioeconomic status 25), and despite the adjustments, women with 5+ live births had a higher cfPWV at visit 5.

In this study, parity was associated with cfPWV at visit 5, but not with cfPWV change. Previous studies have shown that arterial stiffening can be detected even in early childhood, with cfPWV progressing on average 0.2–0.7 m/s every 5 years (26). In older adults, this progression is more rapid, with cfPWV increasing on average by 0.8 m/s per year in those aged over 65 years, and by 0.99 m/s per year in those aged over 70 years (26). No other studies were found that looked at parity and progression of PWV over time. The lack of an association between parity and cfPWV change indicates that the vascular changes associated with pregnancy affect cfPWV earlier in life, but not how arterial stiffness progresses over time in later-life.

The progression of arterial stiffness during pregnancy has been evaluated and has shown that arterial stiffness changes to accommodate the presence and growth of the fetus, with PWV increasing in early pregnancy, decreasing in the second trimester, and returning to the woman's pre-pregnancy baseline in the postpartum period (27). This change is no longer significant when adjusted for MAP, indicating that the change in arterial stiffness is likely a response to the change in blood pressure observed during pregnancy. However, in women with more births, it may be less likely that these vascular alterations will completely return to their pre-pregnancy state, which could result in the differences we observed in later-life PWV. Since PWV in this study was measured much later than pregnancy-related vascular changes, it is possible that age-related progression of arterial stiffness and the prevalence of other vascular risk factors outweighs the effect of parity on cfPWV change. Additionally, because the cfPWV measurements were collected when the participants were 67–88 years of age, it is possible that parity has a more significant impact on arterial stiffness and progression of arterial stiffness earlier in the participants' lives than in later life. Obstetrician-Gynecologists could integrate CVD screening into postnatal care for primary prevention among women with higher parity. In mid-life, women with higher parity should be monitored for CVD risk, consistent with the American Heart Association's CVD prevention guidelines for including obstetric history when evaluating a woman's cardiovascular risk (5). Future work assessing the progression of arterial stiffness earlier in women's lives, particularly during the reproductive lifespan, could help to clarify if other factors in later life outweigh parity's effect on the progression of arterial stiffness or if there is no effect.

The categorical analysis of cfPWV change in this study, defined as cfPWV change >75th percentile, shows that women who were never pregnant or had no live births have a higher likelihood of elevated progression of cfPWV compared to women with 1–2 live births. This finding may be influenced by the effect of miscarriage, stillbirth, or therapeutic abortion on arterial stiffness or conditions that predispose women to pregnancy loss, such as with polycystic ovarian syndrome (PCOS), which is also associated with a higher risk of CVD (28). However, the small number of participants who were previously pregnant and had no live births limits the assessment of pregnancy loss in this analysis.

This study has a few limitations. Information on pregnancy outcomes and complications were not available, which can be more common in certain parity groups (29) and could affect arterial stiffness in these groups to varying degrees, as it is currently unclear what the causative relationship is between arterial stiffness and pregnancy outcomes and complications. Several demographic factors being assessed were self-reported many years after their occurrence and may be affected by recall bias, though recall of reproductive factors (age at menses and age at menopause) are moderately to highly accurate, even years later (30, 31). Additionally, arterial stiffness was only measured in older adulthood, thus we are unable to account for arterial stiffness levels before pregnancy. Lastly, there is the potential for survival bias due to attrition over the course of >25 years of follow-up. The surviving participants are likely healthier than those who did not attend the ARIC visit 5, which may attenuate the observed associations. Our internal validity of the results is supported by our multivariable statistical adjustment for participant characteristics related to attrition. However, it should be noted that this analysis was limited to White and African American participants with African American participants coming from a single study site, so these results may not be generalizable to other racial or ethnic groups or to African Americans in other geographic locations. We were unable to adjust for other social factors, such as lower socioeconomic status, neighborhood disadvantage, and healthcare access, though these factors are strongly related to higher parity and likely coincide with field center and education level, which were adjusted for in this study.

In conclusion, reproductive factors have an important role in evaluating women's cardiovascular risk, but the association of parity with subclinical CVD has not been fully determined. Demonstrating the association between parity and arterial stiffness helps to address this gap in knowledge. It highlights a potential target for primary prevention strategies in women with 5 or more prior live births who do not have clinical CVD. The mechanism by which pregnancy or parity increases arterial stiffness is unclear and requires more longitudinal research among women of younger ages.

## Data Availability

The datasets presented in this study can be found in online repositories. The names of the repository/repositories and accession number(s) can be found below: The data underlying the results presented in the study are publicly available from NHLBI Biologic Specimen and Data Repository (https://biolincc.nhlbi.nih.gov/studies/aric/).
